# Green Onion-Derived Exosome-like Nanoparticles Prevent Ferroptotic Cell Death Triggered by Glutamate: Implication for GPX4 Expression

**DOI:** 10.3390/nu16193257

**Published:** 2024-09-26

**Authors:** Han Jun Yoon, Jun Pil Won, Hyuk Gyoon Lee, Han Geuk Seo

**Affiliations:** Department of Animal Food Resources, College of Sang-Huh Life Sciences, Konkuk University, 120 Neungdong-ro, Gwangjin-gu, Seoul 05029, Republic of Korea; yoon3448@naver.com (H.J.Y.); ww94ww@naver.com (J.P.W.); krci-12@daum.net (H.G.L.)

**Keywords:** green onion-derived exosome-like nanoparticle, ferroptosis, glutamate, iron accumulation, glutathione peroxidase 4, lipid peroxidation

## Abstract

In recent years, alongside research on mammalian-derived exosomes, there has been increasing interest in the physiological activities of plant-derived exosome-like nanoparticles (PDEN). The biocompatibility, minimal side effects, and diverse bioactive ingredients contained in PDEN make them valuable as potential therapeutic agents for an extensive range of diseases. In this study, we cost-effectively isolated exosome-like nanoparticles from green onion (Allium fistulosum) using polyethylene glycol and examined their biological activity in HT-22 cells exposed to glutamate. The isolated green onion-derived exosome-like nanoparticle (GDEN) had an average diameter of 167.4 nm and a zeta potential of −16.06 mV. GDEN effectively inhibited glutamate-induced Ca^2+^ influx and lipid peroxidation, thereby preventing ferroptotic cell death in HT-22 mouse hippocampal cells. Additionally, GDEN reduced the intracellular iron accumulation by modulating the expression of proteins associated with iron metabolism, including transferrin receptor 1, ferroportin 1, divalent metal transporter 1, and ferritin. Notably, GDEN upregulated the expression of glutathione peroxidase 4, a potent antioxidant protein involved in ferroptosis, along with an increase in glutathione synthesis. These findings indicate that GDENs have the potential to serve as bioactives from natural sources against glutamate-induced neuronal cell death, like ferroptosis. This study advances the investigation into the potential medical applications of GDEN and may provide a new approach for the utilization of these bioactive components against neuronal disorders.

## 1. Introduction

Ferroptosis is a newly identified form of programmed cell death, triggered by the iron-mediated peroxidation of lipids [1]. Although it shares similarities with other types of cell death, such as apoptosis and necrosis, ferroptosis differs in that it is caused by the excessive accumulation of iron and lipid peroxides [2]. In the central nervous system, iron is a crucial molecule in various processes, including oxygen transport, myelin production, oxidative phosphorylation, and neurotransmitter metabolism [3]. However, excess iron resulting from abnormal iron homeostasis is stored as ferritin or in labile iron pools, which are not protein-bound, causing cellular damage via the production of hydroxyl radicals. [4]. Ferroptosis has been linked to several pathological conditions characterized by disrupted iron homeostasis, including Parkinson’s diseases and Alzheimer’s [5,6]. In fact, studies in Alzheimer’s model mice show elevated iron levels in neurons [7]. Therefore, modulating ferroptosis may be a strategy to slow disease progression and protect neurons.

Glutamate, a key neurotransmitter in the nervous system, can trigger ferroptosis by disrupting system Xc^−^ [8]. Excess glutamate is known to induce ferroptosis by increasing Ca^2+^ influx and altering iron homeostasis [9,10]. Glutamate-induced iron accumulation increases the generation of reactive oxygen species (ROS) through the oxidization of iron, which causes lipid peroxidation and, ultimately, cell death [1]. In neurons, iron is transported through the blood–brain barrier and into cells via iron channel proteins such as divalent metal transporter 1 (DMT1) and transferrin receptor 1 (TfR1) [3]. Iron is stored mainly as ferritin in cells and is exported out of cells by ferroportin 1 (FPN1). In addition, glutamate inhibits system Xc^−^ and reduces the expression of glutathione peroxidase 4 (GPX4), which lowers cellular glutathione (GSH) levels and impairs the cell’s ability to neutralize ROS, causing oxidative stress [11]. Indeed, increased intracellular glutathione levels and GPX4 expression protect cells by regulating oxidative stress and lipid peroxidation [12].

Exosomes are nanosized (30–200 nm) vesicles composed of a phospholipid bilayer, are secreted by cells, and play a role in intercellular signaling [13,14]. Exosomes contain biomolecules including lipids, proteins, and miRNAs, which are essential for intercellular communication [14,15]. Research about exosomes initially focused on animal cells, which revealed the role of exosomes in a range of pathological and physiological processes, such as immune responses [16], tumorigenesis [17], and neurodegenerative diseases [18]. Exosomes have higher targeting ability than conventional drugs or compounds and can enhance drug delivery, making them promising candidates for next-generation therapeutics [19,20]. Similarly, exosome-like nanoparticles derived from plant cells have also attracted attention as an alternative to animal-derived exosomes because they show higher biocompatibility, are easier to mass-produce, and have a more varied bioactive substance content than mammalian exosomes [21]. The diverse bioactive substance content of plant-derived exosome-like nanoparticles (PDENs) has been reported to elicit a variety of biological activities, such as anticancer [22], antioxidant [23], anti-inflammatory [24], and antimicrobial activities [25]. Because PDENs isolated from different plant species differ considerably in their bioactive compound content, PDENs must be isolated from each plant species to investigate their pharmacological activities. [26]. In this context, we isolated exosome-like nanoparticles from green onion (*Allium fistulosum*), a perennial plant in the onion family (Alliaceae) that is widely cultivated in East Asia, particularly in Republic of Korea, China, and Japan [27]. It is a nutritionally valuable food because of its diverse mineral and vitamin content, such as vitamins A, C, and K [27]. Traditionally, green onion has been used to treat colds, headaches, stomach aches, and cardiovascular diseases [28]. In fact, extracts from green onion inhibit the proliferation of MDA-MB-453 cancer cells and the differentiation of 3T3-L1 adipose progenitor cells [29]. A recent study also found that an ethanolic extract of green onion alleviated non-alcoholic fatty liver disease [30]. However, neither the bioactivities of green onion in neuronal cells nor those of its exome-like nanoparticles have been investigated.

## 2. Materials and Methods

### 2.1. Reagents

L-glutamate and polyethylene glycol 8000 were sourced from Sigma-Aldrich (St. Louis, MO, USA). Monoclonal antibody-targeting DMT1 (dilution 1:1000) was sourced from Santa Cruz Biotechnology (Dallas, TX, USA). Monoclonal antibodies for GPX4 (dilution 1:1000) and ferritin (dilution 1:5000), as well as a polyclonal antibody for TfR1 (dilution 1:1000), were supplied by Abcam (Cambridge, UK). Novus Biologicals (Centennial, CO, USA) provided the polyclonal antibody for FPN1 (dilution 1:1000). Polyclonal antibodies conjugated with horseradish peroxidase for rabbit immunoglobulin G (dilution 1:5000) and mouse immunoglobulin G (dilution 1:10,000) were sourced from Gentex Inc. (Irvine, CA, USA).

### 2.2. Cell Culture

Mouse hippocampal neuronal HT-22 cells were cultivated in Dulbecco′s Modified Eagle′s Medium (DMEM) containing 10% heat-inactivated fetal bovine serum and antibiotics (100 μg/mL streptomycin and 100 U/mL penicillin) at 37 °C in 5% CO_2_.

### 2.3. Isolation of Green Onion-Derived Exosome-like Nanoparticles (GDENs)

GDENs were isolated by a modified polyethylene glycol (PEG) precipitation method which minimizes financial and time expenditures while ensuring high yield and quality of the isolated GDENs [31]. Briefly, green onion was purchased from a local market. Green onion (500 g) was washed thoroughly with phosphate-buffered saline (PBS) and ground in an electric blender for 3 min, with 1 min on/30 s off intervals. The ground green onion was filtered through a cloth filter to remove large debris, and the filtrate was centrifuged sequentially at 3000× *g* for 10 min, 7000× *g* for 20 min, and 12,000× *g* for 30 min to eliminate impurities. The supernatant was filtered using a 0.45 μm syringe filter (Corning, Glendale, AZ, USA). PEG 8000 (Sigma-Aldrich) was then dissolved in the filtrate to achieve a final concentration of 8% and incubated at 4 °C overnight for precipitation. After centrifugation at 7000× *g* for 30 min, the pellet was dissolved in PBS and filtered using a 0.20 μm syringe filter (Corning). The isolated GDENs were aliquoted in 1 mL portions and kept at −80 °C.

### 2.4. Characterization of GDENs

The zeta potential and particle size of the GDENs were investigated with a Zetasizer (Zetasizer Nano ZS90, Malvern Instruments, Malvern, UK) using the electrophoretic light scattering (ELS) and dynamic light scattering (DLS) methods, respectively [32]. To measure the particle size, 1 mL of the GDEN fraction was placed in a disposable cuvette, measured three times, and the mean value was calculated. To measure zeta potential, the GDEN fraction (800 μL) was placed in a folded capillary zeta cell (Malvern Instruments), measured in triplicate, and the mean value was calculated. GDENs morphology was determined using a transmission electron microscope (TEM, JEM 1010, JEOL Ltd., Tokyo, Japan). Briefly, the GDEN fraction (5 μL) was loaded on a formvar carbon-coated grid and allowed to stand for 5 min. Then, 5 μL of phosphotungstic acid was applied to the grid and incubated for 5 min for negative staining. After removal of excess phosphotungstic acid, the grid was dried overnight. To examine the RNA content of the GDENs, total RNA was isolated with TRIzol (Invitrogen, Waltham, MA, USA). The isolated RNA was incubated at 37 °C for 30 min with or without RNase A and then loaded onto a 1% agarose gel.

### 2.5. Cytotoxicity Assay

The effects of GDENs against glutamate toxicity were investigated using the 3-(4,5-dimethylthiazol-2-yl)-2,5-diphenyltetrazolium bromide (MTT) assay and the lactate dehydrogenase (LDH) release assay, following previously established methods [33]. In brief, HT-22 cells were seeded in 35 mm dishes with 10^4^ cells/mL, then incubated for 24 h. The cells were then exposed to glutamate, with or without the addition of 50 μg GDENs, for 16 h. Following replacement with 10% MTT solution, cells were incubated at 37 °C for another 2 h. After removal of all media, isopropanol containing 4 mM HCl was added to solubilize the formazan completely. Absorbance was recorded at a 570 nm wavelength using a Multiskan GO UV-spectrophotometer (Thermo Scientific, Waltham, MA, USA). The LDH release assay was performed using the CytoTox 96 Non-radioactive Cytotoxicity Assay kit (Promega, Madison, WI, USA) as per previously described protocols [33].

### 2.6. Measurements of Calcium Influx and Lipid Peroxidation

Calcium influx and lipid peroxidation were determined by Fluo-3AM and BODIPY fluorescence staining, respectively, as described previously [34]. To assess the calcium influx, 1.16 × 10^5^ cells were seeded in a 35 mm dish and incubated with or without glutamate and/or GDENs for the indicated time. Then, the cells were stained with 200 nM Fluo-3 AM (BD Biosciences, Franklin Lakes, NJ, USA) for 30 min at 37 °C. After staining, cells were detached with trypsin. The fluorescence of the collected cells was measured by a BD FACSCalibur flow cytometer (BD Biosciences). To assess the lipid peroxidation, HT-22 cells that were seeded and treated as described earlier were incubated for the indicated periods. Following incubation, 10 μM BODIPY-C11 (581/591) (Invitrogen) was used for cell staining. The cells were incubated with 10 μM BODIPY-C11 (581/591) for 30 min at 37 °C. The cells were then washed with ice-cold PBS and trypsinized. The fluorescence intension of the collected cells was also analyzed by a BD FACSCalibur flow cytometer (BD Biosciences).

### 2.7. Measurement of Intracellular Iron Levels

Intracellular iron levels in HT-22 cells were determined using a modified ferene-based iron quantification technique, as previously reported [35]. In summary, 7.5 × 10⁵ cells were seeded into 100 mm dishes and incubated with 4 mM glutamate, with or without 50 μg GDENs, for 16 h. Then, incubated cells were lysed in radioimmunoprecipitation assay (RIPA) buffer, followed by sonication (Q55, Qsonica, Newtown, CT, USA). Following centrifugation at 12,000× *g* for 15 min at 4 °C, the amount of iron in the supernatant was measured. To quantify the iron content in the supernatant, 2.5 M ammonium acetate buffer (pH 4.5, 100 μL) and a working solution consisting of 10 mM ascorbic acid and 5 mM ferene in 2.5 M ammonium acetate buffer (pH 4.5, 120 μL) were added to 100 μL of the supernatant. The mixture was left to incubate for 16 h at room temperature, shielded from light. Afterward, 100 μL of the mixture was transferred to a 96-well plate, and absorbance was recorded at 595 nm using a Multiskan GO UV-spectrophotometer (Thermo Scientific). The intracellular iron levels were determined based on a standard iron curve and normalized to the protein concentration.

### 2.8. Total Glutathione Quantification

The intracellular glutathione concentration was analyzed using the glutathione assay kit (BO-GLU-200, Biomax, Guri-si, Republic of Korea). Briefly, 7.5 × 10^5^ cells were seeded into a 100 mm culture dish and incubated with 4 mM glutamate with or without 50 μg GDENs for 16 h. After removing all the substances with ice-cold PBS, the cells were trypsinized and the cell number was counted. Then, 2 × 10^6^ cells were lysed in 200 μL 5% metaphosphoric acid (MPA) and homogenized by sonication. After centrifugation at 13,500× *g* for 10 min, the supernatant was used for further experiments. Oxidized glutathione (GSSG) in the supernatant was reduced with glutathione reductase, and 5,5’-dithiobis 2-nitrobenzoic (DTNB) was added. For 5 min with 30 s intervals, the absorbance was measured at 412 nm and the absorbance change was measured. The slope of the absorbance change was determined using a slope of the GSSG standard.

### 2.9. Western Blot

HT-22 cells (7.5 × 10⁵) were plated in 100 mm culture dishes and incubated with 4 mM glutamate, with or without 50 μg GDENs. The cells were rinsed twice to remove the remaining media with ice-cold PBS and collected with a cell scraper. After centrifugation at 16,000× *g* for 1 min, the cell pellet was lysed using PRO-PREP Protein Extraction Solution (iNtRON Biotechnology, Seoul, Republic of Korea), and 30 μg of the protein extract was separated by SDS-polyacrylamide gel electrophoresis. The proteins were subsequently transferred onto an Immobilon-P polyvinylidene difluoride membrane (Merck, Darmstadt, Germany). Then, the membrane was incubated in 5% non-fat milk dissolved in Tris-buffered saline containing 0.1% Tween-20 (TBS-T) for 16 h at 4 °C for blocking, followed by a 30 min wash in TBS-T, along with replacement with fresh TBS-T every 10 min to remove excess antibodies that had been washed away. Subsequently, the blocked membranes were incubated overnight at 4 °C with the primary antibody. After a 30 min wash in TBS-T, the membrane was soaked in the secondary antibody, which was diluted in 5% non-fat milk dissolved in TBS-T for 1 h at room temperature. Following another 30 min wash with TBS-T, chemiluminescence was detected using the WesternBright ECL solution (Advansta Inc., San Jose, CA, USA).

### 2.10. Statistical Analysis

Statistical analyses were conducted using the Statistical Package for the Social Sciences (SPSS, Version 25; IBM Corp., New York, NY, USA). Data are expressed as means ± standard error. Statistical significance was assessed using Student’s *t*-test or one-way ANOVA, followed by Tukey’s post hoc test if the data followed normality or non-parametric statistics if the data did not follow normality.

## 3. Results

### 3.1. Isolation and Characterization of GDENs

**Figure 1:**
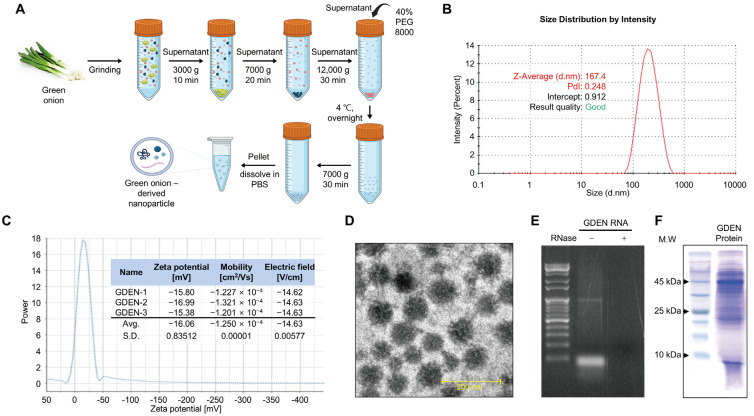
Isolation and characterization of GDENs. (**A**) Schematic presentation of the polyethylene glycol precipitation method used to isolate GDENs from green onions. (**B**–**F**) Characteristics of GDENs. (**B**) Determination of GDEN size using dynamic light scattering (DLS). (**C**) Zeta potential of GDENs. (**D**) Transmission electron microscopy image of GDENs. (**E**) The size distribution of proteins from GDENs. (**F**) Size distribution of total RNA from GDENs. The RNA extract of GDENs was treated with or without RNase A.

GDENs were obtained by performing PEG precipitation of ground green onion extracts after differential centrifugation to remove large debris and the pellet (Figure 1A). To characterize the isolated GDENs, particle size and zeta potential were analyzed. DLS analysis and zeta potential measurements revealed that the GDENs had an average particle size of 167.4 nm and a zeta potential value of −16.06 mV (Figure 1B,C). TEM images showed that the GDENs had a uniform and round shape, with a diameter of less than 200 nm (Figure 1D). Total RNA electrophoresis was performed to identify RNAs, which are characteristic components of plant-derived exosome-like nanoparticles (PDENs). The electrophoresis revealed bands of 100 bp or less. RNase treatment resulted in the complete disappearance of the bands, demonstrating that they contained RNA (Figure 1E). Additionally, SDS-polyacrylamide gel electrophoresis of the GDEN protein extracts revealed proteins with a wide range of molecular weights (Figure 1F). And the concentration of the isolated GDENs was 4.45 μg/μL.

### 3.2. GDENs Inhibit Glutamate-Induced Ferroptotic Cell Death in HT-22 Cells

**Figure 2:**
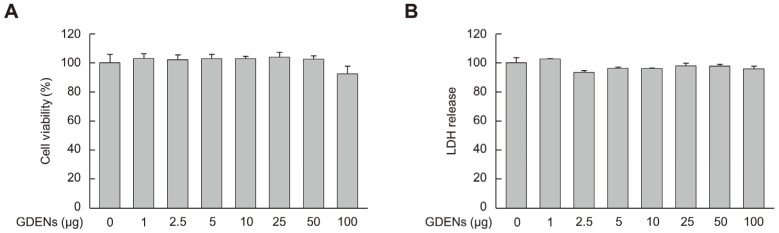
Cellular toxicity of GDENs was evaluated in HT-22 mouse hippocampal neurons. Cells were exposed to GDENs at specified concentrations for 24 h. (**A**) The MTT assay was used to assess cell viability. (**B**) The LDH release assay was used to assess cellular toxicity. Results are expressed as mean ± SE (*n* = 3).

To determine the appropriate concentrations of GDENs for subsequent experiments, MTT and LDH release assays were performed using HT-22 cells to measure cell viability and cell toxicity, respectively. Although cellular toxicity was not observed, cell viability tended to decrease at 100 μg GDENs, although the decrease was not significant (Figure 2A,B). Thus, we used 50 μg GDENs in subsequent experiments. 

**Figure 3:**
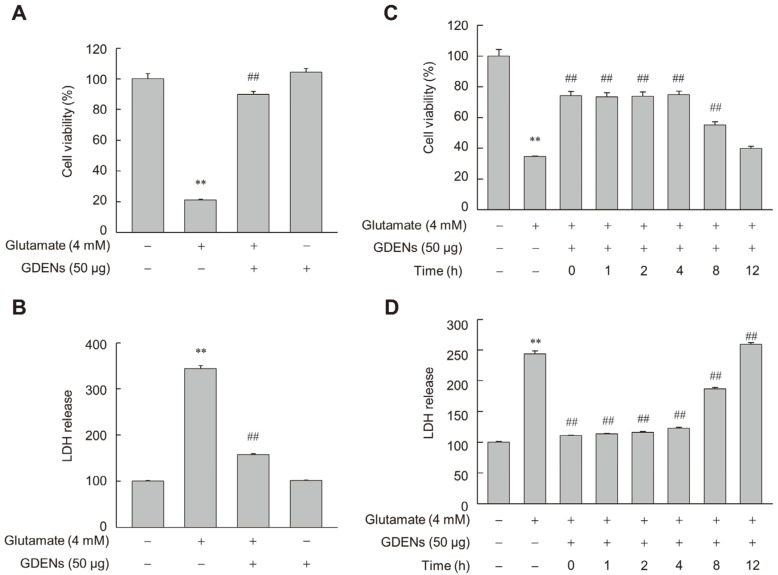
GDENs inhibit glutamate-induced cell death in HT-22 cells. (**A**,**B**) Cells were exposed to 4 mM glutamate for 16 h, with or without the addition of 50 μg GDENs. Cell viability was assessed using the MTT assay (**A**), and cellular toxicity was evaluated with the LDH release assay (**B**). (**C**,**D**) Following a 16 h treatment with 4 mM glutamate, cells were treated with 50 μg GDENs for the specified durations. The times indicate when the GDENs were treated after glutamate treatment. The MTT assay was performed (**C**), and the LDH release assay was conducted (**D**). Results are shown as mean ± SE (*n* = 3). ** *p* < 0.01 compared to the control group; ## *p* < 0.01 compared to the glutamate-treated group.

To assess whether GDENs inhibit glutamate-induced ferroptotic cell death in HT-22 cells, we performed MTT and LDH release assays after incubating the cells with 4 mM glutamate with or without 50 μg GDENs (Figure 3A,B). Furthermore, GDENs conferred significant cytoprotective effects even when added 8 h post-treatment with glutamate (Figure 3C,D).

### 3.3. GDENs Inhibit Glutamate-Induced Calcium Influx

**Figure 4:**
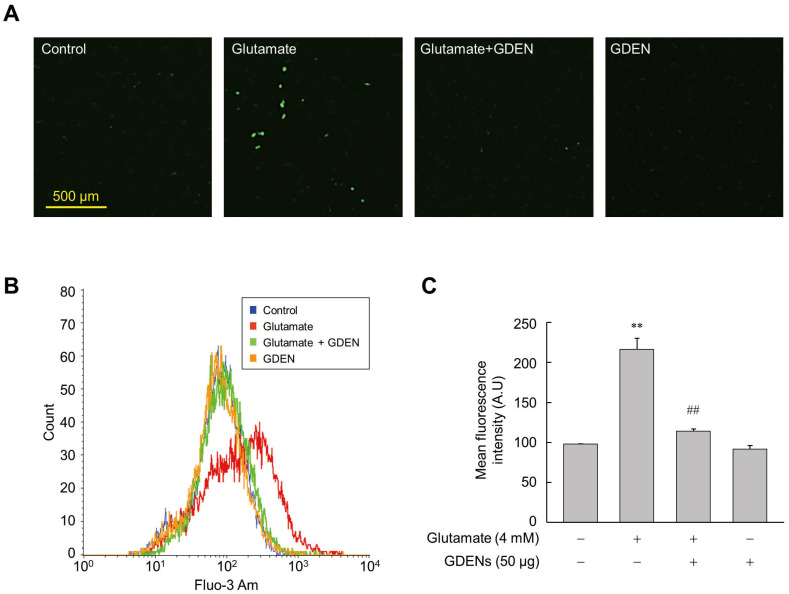
GDENs suppress glutamate-triggered Ca^2+^ influx. (**A**–**C**) HT-22 cells were exposed to 4 mM glutamate for 16 h with or without 50 μg GDENs. Fluorescence microscopy image of Fluo-3 Am staining (**A**). Intracellular Ca^2+^ levels were assessed by FACS (**B**) and quantified by measuring the mean fluorescence intensity of Fluo-3 Am (**C**). The results are shown as mean ± SE (*n* = 3). ** *p* < 0.01 compared to the control group; ## *p* < 0.01 compared to the glutamate-treated group.

Intracellular calcium influx was recognized as one of the features that precedes cell death induced by ferroptosis [9]. To investigate whether GDENs inhibit intracellular calcium influx, we utilized Fluo-3AM fluorescent staining. Fluorescence microscopic analysis revealed that glutamate treatment increased calcium influx into the cells, as indicated by the elevated fluorescence of Fluo-3AM, while co-treatment with GDENs reduced glutamate-induced calcium influx (Figure 4A). For more accurate quantification, we employed flow cytometry to measure the mean fluorescence intensity of Fluo-3AM. As is consistent with the results of fluorescence microscopy, the results obtained from flow cytometry confirmed that treatment with GDENs attenuated glutamate-induced calcium influx in HT-22 cells significantly (Figure 4B,C).

### 3.4. GDENs Attenuate the Labile Iron Pool by Regulating Iron Metabolism-Related Proteins

**Figure 5:**
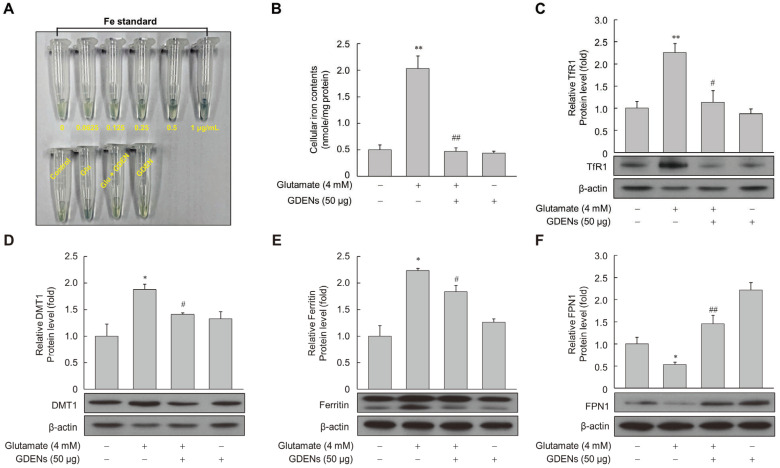
GDENs reduce intracellular iron levels by regulating the iron metabolism-related protein expression. (**A**–**F**) HT-22 cells were exposed to 4 mM glutamate for 16 h, with or without the addition of 50 μg GDENs. Intracellular iron levels were measured using the ferene-based colorimetric method (**A**,**B**). Protein expression was evaluated via Western blot (**C**–**F**), and β-actin was used as an internal control. Results are presented as mean ± SE (*n* = 3). * *p* < 0.05, ** *p* < 0.01 compared to the control group; # *p* < 0.05, ## *p* < 0.01 compared to the glutamate-treated group.

To determine whether GDENs protect against glutamate-induced disruption of iron homeostasis, cellular iron levels were measured using a ferene-based iron quantification method. While intracellular iron levels were significantly increased after glutamate treatment, GDENs effectively reversed the effects of glutamate treatment (Figure 5A,B). Several proteins which are related to intracellular iron metabolism have been identified, including transferrin receptor 1 (TfR1), ferroportin 1 (FPN1), divalent metal transporter 1 (DMT1), and ferritin. Western blot analysis of these iron metabolism-related proteins showed that their levels were increased by glutamate treatment and reversed by co-treatment with GDENs (Figure 5C–E). On the other hand, the glutamate-induced decrease in FPN1 expression was reversed by co-treatment with GDENs (Figure 5F).

### 3.5. GDENs Suppress Glutamate-Induced Lipid Peroxidation by Up-Regulating GPX4 Expression

**Figure 6:**
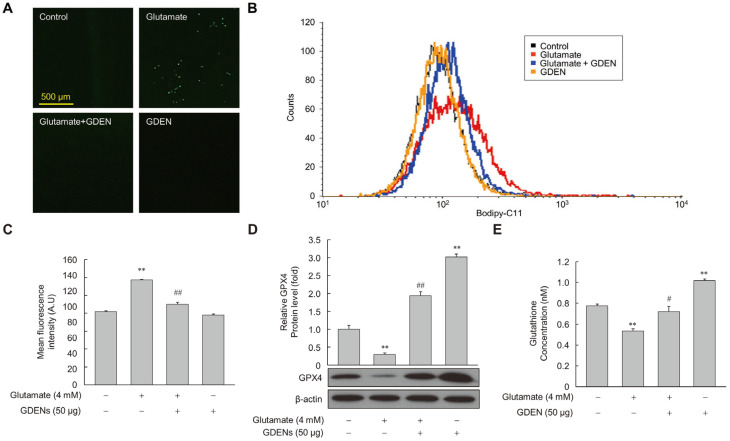
GDENs suppress glutamate-induced lipid peroxidation. (**A**–**E**) HT-22 cells were exposed to 4 mM glutamate for 6 h, with or without 50 μg GDENs. Fluorescence microscopy images of Bodipy-C11-stained cells were captured (**A**). Lipid peroxide levels were assessed using FACS (**B**) and quantified by assessing the mean fluorescence intensity of Bodipy-C11 (**C**). GPX4 levels were evaluated by Western blot and quantified using ImageJ (version 1.53t) (**D**), with β-actin as an internal control. Intracellular glutathione concentration was analyzed by the glutathione assay kit, and the concentration in the graph represents the glutathione concentration per cell (**E**). Results are shown as mean ± SE (*n* = 3). ** *p* < 0.01 compared to the control group; # *p* < 0.05, ## *p* < 0.01 compared to the glutamate-treated group.

To assess whether GDENs inhibited lipid peroxidation induced by glutamate, we measured the amount of peroxidized lipids using Bodipy-C11 fluorescence staining after glutamate treatment with or without GDENs. Under fluorescence microscopy, we observed that fluorescence intensity was higher in the glutamate-treated group than in the control group, but it decreased markedly upon co-treatment with GDENs (Figure 6A), indicating that GDENs inhibited glutamate-induced lipid peroxidation. The results were confirmed more accurately by measuring fluorescence intensity by flow cytometry (Figure 6B,C). Moreover, we investigated the expression of GPX4. Our results demonstrated that GDEN treatment significantly reversed the glutamate-induced decrease in the expression of GPX4, a major antioxidant enzyme involved in ferroptosis (Figure 6D). Furthermore, GDEN treatment significantly increased the synthesis of glutathione, a critical antioxidant that functions as a substrate of GPX4 to prevent lipid peroxidation (Figure 6E). These results suggest that GDEN-mediated protection against glutamate-induced ferroptosis may be mediated, at least in part, by the upregulation of GPX4 expression and increase in glutathione synthesis, thereby decreasing lipid peroxidation and cell death.

## 4. Discussion

This study shows that GDENs prevent cell death by inhibiting ferroptosis in mouse hippocampal HT-22 cells. We cost-effectively isolated GDENs using a PEG precipitation method. The isolated GDENs showed a rounded shape with an average diameter of 167.4 nm and a stable mean zeta potential of −16.06 mV. These properties were similar to those of exosomes isolated using sucrose gradient ultracentrifugation, a conventional exosome isolation method [36], indicating that PEG precipitation is an effective method for the preparation of GDENs. GDENs significantly inhibited glutamate-induced ferroptosis by regulating iron metabolism, GPX4 expression, and increasing glutathione synthesis, which are major regulatory factors of ferroptosis. These observations suggest that GDEN formulations could serve as a potential alternative treatment for the prevention and treatment of glutamate-related disorders in neurons.

Exosome-like nanoparticles from diverse plants are typically characterized by their shape, size, surface charge, and composition [37]. In general, PDENs isolated by ultracentrifugation have particle diameters ranging from tens to hundreds of nanometers and zeta potentials ranging from −0.6 mV to −34 mV [38,39]. As is consistent with previous reports, GDENs isolated by PEG precipitation had uniform, rounded shapes with diameters in the range of 30–200 nm and a stable zeta potential of −16.06 mV, demonstrating that they are stably dispersed in the colloidal state. In addition, GDENs were composed of proteins and small RNAs of various sizes, which are the characteristics of PDEN, in line with the characteristics of PDENs in previous reports [40]. Small RNAs in exosomes from mammalian cells have been shown to have multiple bioactivities, including anti-inflammatory and anticancer effects [41,42]. Considering the physiological and pharmacological activities of small RNAs from plant-derived exosomes [43,44], the small RNAs in GDENs may have also contributed to their biological activities in this study.

It is widely recognized that Ca^2+^ influx is linked with cytotoxicity and cell death [45]. A previous study reported that glutamate induces Ca^2+^-dependent cell death by inhibiting cystine influx through the cystine/glutamate reverse transporter system Xc^−^ [46]. Indeed, previous studies have found that glutamate-treated HT-22 cells are resistant against apoptosis when cultured in Ca^2+^-free medium or treated with cobalt, a calcium channel inhibitor [47]. In addition, increased intracellular Ca^2+^ levels can preferentially sensitize refractory cancer cell lines to ferroptosis [48]. A crosstalk model for the relationship between Ca^2+^ and iron in ferroptosis has recently proposed that iron-mediated excessive ROS production disrupts Ca^2+^ homeostasis, generating a vicious cycle in which excess Ca^2+^ contributes to increased intracellular iron levels [49]. As is consistent with the previous study, our results showed that the glutamate-triggered increase in intracellular Ca^2+^ in HT-22 cells was significantly reduced by GDENs. This observation indicates that GDENs could be used to inhibit neuronal cell death by preventing increases in Ca^2+^ levels in neurons, with effects on downstream signaling pathways, such as those mediating intracellular iron accumulation.

Iron chelators like deferoxamine (DFO) can inhibit ferroptosis, indicating the importance of intracellular iron levels in ferroptosis [1]. Several proteins involved in iron metabolism have been identified as key factors in ferroptosis. In fact, GW501516, a peroxisome proliferator-activated receptor δ agonist, inhibits ferroptosis by reducing intracellular iron levels [33]. As indicated in previous reports, it is crucial to assess whether altering the expression of iron metabolism-related proteins can control ferroptosis [7]. In this study, we demonstrated that GDENs decreased the glutamate-induced expression of iron influx-related proteins DMT1 and TfR1 and the iron release-related protein FPN1, leading to reduced intracellular iron levels in glutamate-treated HT-22 cells. These results indicate that GDENs ameliorate ferroptosis by inhibiting excessive intracellular iron accumulation induced by glutamate via the regulation of the expression of proteins involved in iron metabolism.

The mechanism by which GPX4 prevents cell death by regulating lipid peroxidation via the system Xc^−^/GSH/GPX4 axis has been well studied, and GPX4 abnormalities are known to induce ferroptosis [1,12]. Indeed, treatment with RSL3, a GPX4 inhibitor, induces ferroptosis in glioblastoma cells [50]. Additionally, the ablation of GPX4 in the mouse forebrain accelerates neurodegeneration and impairs cognitive function induced by ferroptosis [51]. Therefore, regulation of GPX4 expression is now considered a key factor in the regulation of ferroptosis. A previous study reported that baicalein suppresses oxygen glucose deprivation/re-oxygenation (OGD/R)-induced ferroptosis by regulating the expression of GPX4 in HT-22 cells [52]. Similarly, we also found that GDENs dramatically attenuated the decrease in glutamine-induced GPX4 expression, resulting in a significant increase in the synthesis of glutathione, a critical antioxidant that works synergistically with GPX4 to prevent lipid peroxidation. These results indicate that GDENs can increase the resistance of cells to ferroptosis by boosting antioxidant defense mechanisms.

Green onions contain sulfur compounds such as allicin, alliin, and ajoene, which have anti-inflammatory effects, along with flavonoids like quercetin and kaempferol, known for their antioxidant properties [27]. The beneficial effects of GDENs could be derived from these secondary metabolites. GDENs may share these components with green onions. In fact, the ferroptosis-inhibiting effects of these secondary metabolites have been demonstrated in several studies. Quercetin and kaempferol have been shown to inhibit ferroptosis via the SIRT1/NRF2/SLC7A11/GPX4 pathway [53,54]. Additionally, ascorbic acid, abundant in green onions, is a well-known antioxidant, and its inhibition of lipid peroxidation in ferroptosis may also contribute to the effects of GDENs. Recent research has indicated that various inflammation-related signaling pathways are linked to ferroptosis [55]. The anti-ferroptotic effects of GDENs may also be attributed to the anti-inflammatory properties of sulfur-containing compounds. However, further studies are needed for a deeper understanding of the mechanism of GDEN’s inhibition of ferroptosis, along with an analysis of its components.

In addition to the pharmacodynamic behavior of GDENs, their pharmacokinetic activity is also important to understand their properties in humans. As expected, GDENs can undergo several metabolic processes in the body just like regular foods; however, due to their nano-sized size, GDENs may be absorbed differently compared to regular foods with larger particles. In fact, Choi et al. has demonstrated that orally administered exosomes are delivered to the brain [56], and a recent study has shown that exosomes can cross the blood–brain barrier [57]. Once delivered to the brain, GDENs are expected to modulate a variety of biological pathways, particularly those related to oxidative stress. While the safety of GDENs has not yet been validated, plant-derived exosomes are known to be relatively biocompatible [21]. However, their long-term safety in preclinical and clinical studies has not yet been established. Therefore, further studies are needed to clarify the safety of GDENs.

## 5. Conclusions

The present study demonstrated that GDENs significantly inhibited glutamate-induced ferroptosis by regulating the expression of iron-metabolizing proteins, GPX4 expression, and glutathione synthesis. These findings support the potential use of GDENs as safe and natural substances for the treatment of disorders caused by ferroptosis. Based on these observations, further research could ultimately aim to develop strategies that target ferroptosis.

## Data Availability

The original contributions presented in the study are included in the article; further inquiries can be directed to the corresponding author due to time limitations.
